# Jiajiejian gel ameliorates thyroid nodules through regulation of thyroid hormones and suppression of the (IL-6, TNF-α, IL-1β)/JAK2/STAT3/VEGF pathway

**DOI:** 10.3389/fphar.2024.1483686

**Published:** 2024-10-18

**Authors:** Changlin Wang, Xiangju Gao, Mingqi Qiao, Dongmei Gao, Yinghui Guo, Jieqiong Wang, Chunhong Song

**Affiliations:** ^1^ Laboratory Animal Center, Central Hospital Affiliated to Shandong First Medical University, Jinan, China; ^2^ School of Chinese Medicine, Shandong University of Traditional Chinese Medicine, Jinan, China; ^3^ School of Pharmacy, Shandong University of Traditional Chinese Medicine, Jinan, China

**Keywords:** Jiajiejian gel, thyroid nodules, external treatment, thyroid hormones, transcriptomics, (IL-6, TNF-α, IL-1β)/JAK2/STAT3/VEGF pathway

## Abstract

**Background:**

The high incidence of thyroid nodules and their rapid growth in recent years have become an important issue affecting public health. Traditional Chinese medicine (TCM) external treatments have unique advantages in treating this disease, but the currently available external preparations are relatively few and the therapeutic mechanism is unclear. Jiajiejian gel (JJJG) is a TCM external preparation developed by our team for the thyroid nodule treatment, which has been preliminarily proven to be safe and effective in clinical practice.

**Objective:**

The current study was aimed to elucidate the therapeutic effects and the underlying mechanisms of JJJG on thyroid nodules in rats.

**Methods:**

The contents of paeonol and forsythoside A in JJJG were determined by HPLC. The thyroid nodules rat model was established through oral gavage of 0.1% propylthiouracil (PTU) for 6 weeks and meanwhile the rats were treated with external JJJG (0.26, 0.52, 1.04 g/kg). Subsequently, the therapeutic effect of JJJG was observed by means of ultrasonic examination, morphology observation, organ coefficients determination and histopathological analysis. Mechanismlly, the levels of FT3, FT4 and TSH in serum were measured and transcriptomics methods were used to analyse and screen the key targets and pathways of alleviating thyroid nodules by JJJG. Further, gene and protein expression levels of key factors in the pathways were measured and validated using quantitative real-time PCR, ELISA, western blotting and immunofluorescence, so as to clarify the therapeutic mechanism.

**Results:**

The contents of the paeonol and forsythoside A were 1.160 and 0.608 mg/g, respectively. JJJG reduced thyroid swelling, improved nodular lesions, decreased thyroid coefficients, and inhibited abnormal nodular hyperplasia of follicular epithelial cells. In terms of mechanism, JJJG significantly increased the levels of FT3 and FT4 and decreased TSH level in serum (*P* < 0.05). Transcriptomics suggested that the (IL-6, TNF-α, IL-1β)/JAK2/STAT3/VEGF pathway may be one of the key mechanisms in the treatment of thyroid nodules by JJJG. Further validation experiments demonstrated that JJJG significantly reduced the mRNA expression and protein content of IL-1β, IL-6 and TNF-α in thyroid tissue, as well as the mRNA expression of JAK2, STAT3 and VEGF and the protein expression of p-JAK2/JAK2, p-STAT3/STAT3 and VEGF (*P* < 0.05).

**Conclusion:**

This study indicates that JJJG efficiently ameliorates thyroid nodules by regulating the levels of FT3, FT4 and TSH in serum and suppressing (IL-6, TNF-α, IL-1β)/JAK2/STAT3/VEGF pathway in thyroid tissue, providing a potential therapeutic approach for thyroid nodules.

## Introduction

Thyroid nodules are discrete lesions caused by abnormal, focal growth of thyroid cells within the thyroid gland (Fernandez et al., 2024). With the improvement of medical conditions and various imaging techniques, the detection rate of thyroid nodules is also increasing (Grani et al., 2020). The 2023 Guidelines for the Diagnosis and Management of Thyroid Nodules and Differentiated Thyroid Cancer (Second Edition) state that the prevalence of thyroid nodules in adults is as high as 20.43% (Yin et al., 2023; Li et al., 2020). The high incidence of thyroid nodules and their rapid growth in recent years have become an important issue affecting public health, bringing huge economic and psychological burdens to patients and seriously jeopardising people’s quality of life (Haugen et al., 2016).

Currently, thyroid nodules can be treated clinically with surgery, radioactive iodine therapy, ultrasound-guided ablation therapy and thyroid hormone suppression therapy (Durante et al., 2018; Cervelli et al., 2019; Sdano et al., 2005). Such methods have certain advantages, especially in laser ablation, which is less invasive and more effective (Zufry and Hariyanto, 2024). However, their disadvantages should not be ignored, such as large adverse reactions, high therapeutic risks, limited application scope, and expensive costs. With the advantages of good efficacy, high safety and wide applicability, the traditional Chinese medicine (TCM) external treatment has unique advantages in alleviating superficial diseases such as thyroid nodules. It can avoid the stimulation of the gastrointestinal tract caused by long-term oral administration and is more easily accepted by patients (Li and Huang, 2023). However, relatively few external pharmaceutical preparations are currently available for the treatment of thyroid nodules, so there is an urgent need for a safe, effective, easy-to-use and aesthetically pleasing external preparation to meet the huge medical and social demand. Jiajiejian gel (JJJG) is a TCM external preparation developed by our team for the treatment of thyroid nodules, the detailed composition of the drug is shown in Table 1. Preliminary results of small scale clinical observations conducted under the principle of informed consent have been obtained, the results of which proved the safety and efficacy of the drug (Qiao et al., 2023). Therefore, it is necessary to further explore its pharmacological effect and mechanism in the laboratory.

**TABLE 1:** The compositions of Chinese medicine in JJJG.

Chinese name	Latin name	Scientific name	Medicinal parts
Mu Dan Pi	*Moutan Cortex*	*Paeonia suffruticosa* Andr	Root bark
Lian Qiao	*Forsythiae Fructus*	*Forsythia suspensa* (Thunb.)	Fruit
Chai Hu	*Bupleuri Radix*	*Bupleurum chinense* DC. and *Bupleurum scorzonerifolium* Willd	Root
Xia Ku Cao	*Prunellae Spica*	*Prunella vulgaris* L	Fruit ear
Huang Qin	*Scutellariae Radix*	*Scutellaria baicalensis* Georgi	Root
Mu Li	*Ostreae Concha*	*Ostrea gigas* Thimberg, *Ostrea talienwhanensis* Crosse and *Ostrea rivularis* Gould	Shell
E Zhu	*Curcumae Rhizoma*	*Curcuma phaeocaulis* VaL., *Curcuma kwangsiensis* S. G. Lee et C. F. Liang and *Curcuma wenyujin* Y. H. Chen et C. Ling	Rhizome
Zhe Bei Mu	*Fritillariae Thunbergii Bulbus*	*Fritillaria thunbergii* Miq	Bulb
Bing Pian	*Borneolum*	*Cinnamomum camphora* (L.) Presl	Branch and leaf

In addition, current reports on the treatment of thyroid nodules with TCM mainly focus on clinical efficacy observation of the drugs, with fewer studies exploring the action mechanism, and the signaling pathways are mainly centred around the hypothalamic-pituitary-thyroid (HPT) axis, the phosphatidylinositol 3-kinase and protein kinase B (PI3K-Akt) pathway and the apoptosis-related pathways, etc (Wang et al., 2023a; Yi, 2010; Li et al., 2017a). Due to the pathogenesis complexity of thyroid nodules, there are multiple related genes and signal transduction pathways that co-regulate the disease. Therefore, revealing more regulatory genes and pathways and intervening their regulatory processes may open up a new avenue for the treatment of thyroid nodules.

**FIGURE 1:**
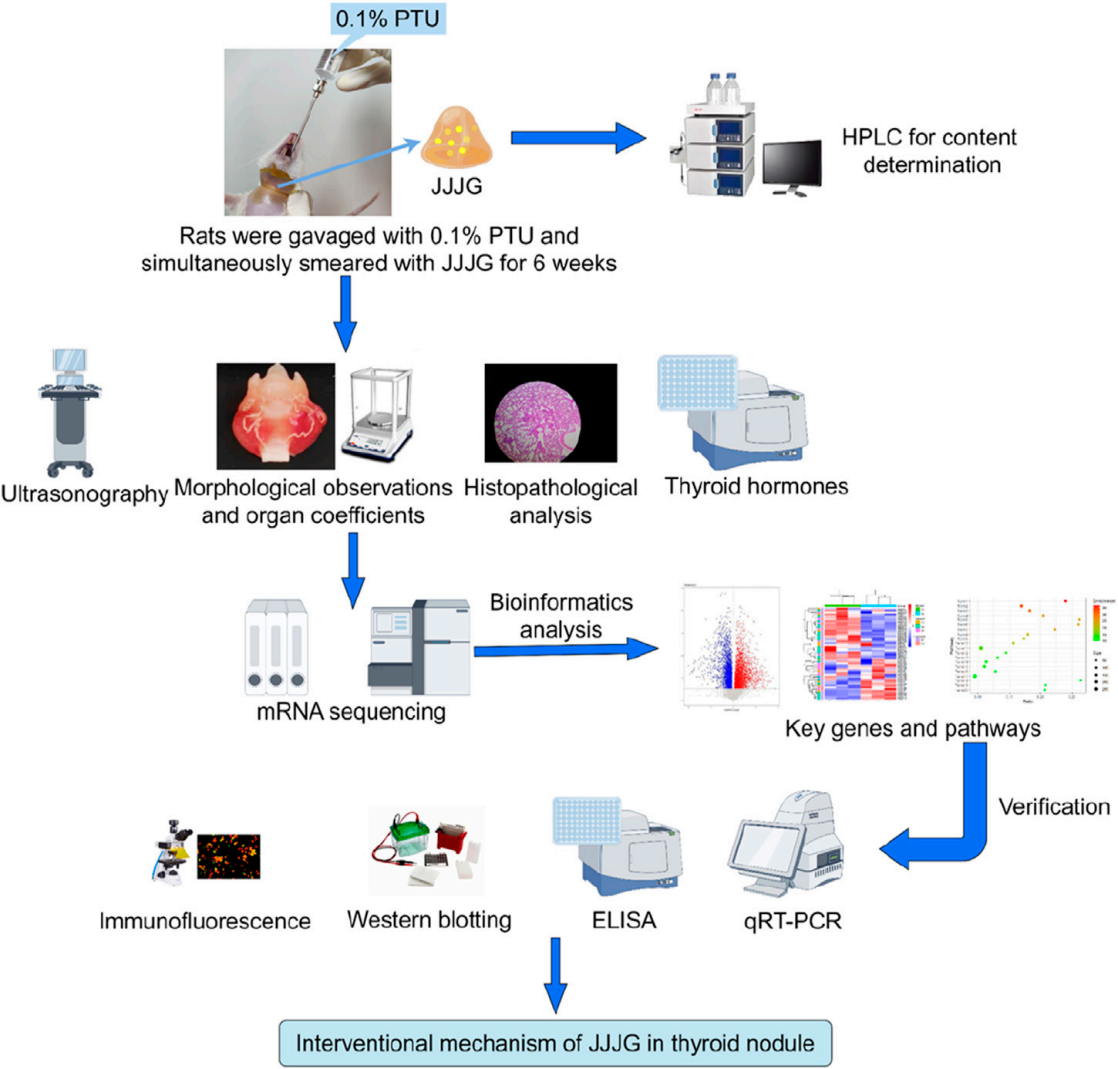
Flowchart outlining the experimental design and methodology of this study.

In this study, the content of active components in JJJG was first determined, and then the therapeutic effect of JJJG on thyroid nodules model rats was investigated by ultrasonography, morphological observation and histopathological analysis. Mechanismally, the levels of thyroid related hormones in serum were measured and transcriptomics methods were used to analyse and screen the key targets and pathways of alleviating thyroid nodules by JJJG. Further, gene and protein expression levels of key factors in the pathways were measured and validated using a series of molecular biology methods to clarify the therapeutic mechanism of JJJG (Figure 1). The initiative can provide reference for the future research and development of clinical external therapeutic drugs for thyroid nodules, highlighting the unique advantages of external therapeutic methods in Chinese medicine, and is of great significance for promoting the TCM modernization.

## Materials and methods

### Drugs and reagents

All medicinal herbs contained in JJJG were purchased from Baisuitang Traditional Chinese Medicine Co., Ltd. (Anhui, China). Propylthiouracil tablets were purchased by Zhaohui pharmaceutical Co., Ltd. (Shanghai, China). Jianingye (JNY) was purchased by Juhetang Medical equipment Co., Ltd. (Henan, China). Primary antibodies against janus kinase 2 (JAK2), signal transducer and activator of transcription 3 (STAT3), p-STAT3 (Tyr705), vascular endothelial growth factor (VEGF) were purchased from Bioss (Wuhan, China). The primary antibody against p-JAK2 was purchased from Abclonal (Wuhan, China). Free triiodothyronine (FT3), free tetraiodothyronine (FT4), interleukin-6 (IL-6), interleukin-1β (IL-1β) and tumor necrosis factor-α (TNF-α) assay kits were provided by Cusabio biological engineering Co., Ltd. (Wuhan, China). Thyroid stimulating hormone (TSH) assay kit was provided by Bioswamp biological technology Co., Ltd. (Wuhan, China). Paeonol (purity 99.97%) and forsythoside A (purity 98.33%) were provided by Must biological technology Co., Ltd. (Chengdu, China).

### Preparation of JJJG

*Moutan Cortex* (Mu Dan Pi) was extracted by steam distillation with 12 times distilled water, and about 6 times distilled liquid was collected at a speed of 2 ± 0.25 BV/h. The drug crystal was obtained by refrigerating the distillate for 12 h, filtering and vacuum drying at 45°C for 1.5 h (Xie et al., 2019). The drug crystal was mixed with a prescription proportion of *Borneolum* (Bing Pian) and fully ground, and then a small amount of 95% ethanol was added to dissolve it to obtain solution A.

*Forsythiae Fructus* (Lian Qiao), *Prunellae Spica* (Xia Ku Cao), *Scutellariae Radix* (Huang Qin), *Bupleuri Radix* (Chai Hu) and *Ostreae Concha* (Mu Li) in prescribed proportions were extracted with 8 times amount of distilled water through heating reflux three times, each time for 1 h. Concentrate the decoction to 1 g crude drug/mL, then add 95% ethanol to make the solution ethanol concentration reach 70% for alcohol precipitation, rest at 4°C overnight, and centrifuge to get the supernatant B.

*Curcumae Rhizoma* (E Zhu) and *Fritillariae Thunbergii Bulbus* (Zhe Bei Mu) were extracted with 6 times amount of 60% ethanol through heating reflux twice, each time for 1.5 h. Filter and merge the filtrate to get the supernatant C. The supernatant B was mixed with C, concentrated to a certain extent, and then solution A was added, then 95% ethanol was added to fix the volume, and finally the drug extract with a concentration of 1 g crude drug/mL was obtained.

1.5% carbomer 940 was swelled with 15 times the amount of distilled water for 12 h, then triethanolamine was added to adjust pH to 7.5, and the matrix I was obtained after further aging for 12 h. The 40% drug extract was fully mixed with 10% glycerol, 0.5% Tween 80, 2% azone, 2% peppermint oil and 0.1% ethylparaben to obtain composition II. The matrix I and composition II were mixed, fully stirred and distilled water was added to make up the proportion to obtain JJJG.

### Content determination of JJJG

The main components of JJJG were analyzed by high performance liquid chromatography (HPLC). 2 g JJJG was added to 10 mL methanol, sonicated for 30 min at 35 kHz and 25°C, and then centrifuged at 3,500 rpm for 5 min. The supernatant was filtered through a 0.22 μm filter before HPLC analysis. The HPLC analysis was carried out by Agilent 1260 HPLC system with Agilent 5 HC-C_18_ column (250 × 4.6 mm, 5 µm). The flowrate was 1.0 mL/min, the injection volume was 10 μL, and the column temperature was maintained at 30°C. The mobile phase, elution programs and detection wavelengths of paeonol and forsythoside A were different. The mobile phase of paeonol consisted of 0.2% phosphoric acid (A)-acetonitrile (B). The elution program of paeonol was as follows: 0–10 min 87%–84% A; 10–20 min 84%–83% A; 20–60 min 83%–34% A; 60–65 min 34%–87% A; 65–70 min 87% A. The detection wavelength of paeonol was 274 nm. The mobile phase of forsythoside A consisted of 0.4% glacial acetic acid (A)- methanol (B). The elution program of forsythoside A was as follows: 0–10 min 75%–66% A; 10–17 min 66%–70% A; 17–35 min 70% A; 35–45 min 70%–25% A; 45–50 min 25%–75% A; 50–60 min 75% A. The detection wavelength of forsythoside A was 330 nm.

### Experimental animals and drug administration

Wistar rats (half male and half female; 6–8 weeks old; weighing 180–220 g) were purchased from Vital River Laboratory Animal Technology Co., Ltd. (Beijing, China). The animals were housed under specific pathogen-free (SPF) conditions (12 h light/dark cycle, 23°C ± 2°C with a relative humidity of 45% ± 10%) with free access to autoclaved food and water in the laboratory animal center of Central Hospital Affiliated to Shandong First Medical University. All procedures and experiments carried out here were approved by the Ethics Review Committee for Animal Experimentation of Central Hospital Affiliated to Shandong First Medical University (Approval NO. JNCHIACUC 2022-60).

After 1 week of acclimatization, 84 rats were stochastically divided into six groups (n = 14/group, half male and half female): control group, model group, low-dose JJJG group (JJJG-L), medium-dose JJJG group (JJJG-M), high-dose JJJG group (JJJG-H), and Jianingye positive control group (JNY). Except for the rats in control group which were gavaged with an equal volume of saline, the rats in the other five groups were intragastric with 0.1% PTU solution at the dose of 1 mL/100 g body weight once per day for 6 weeks (Pei et al., 2022; Li, 2018). At the same time, according to our previous study, the rats in each treatment group was smeared with the corresponding dose of drugs per day for six consecutive weeks, namely, JJJG-L (0.26 g/kg), JJJG-M (0.52 g/kg), JJJG-H (1.04 g/kg) and JNY (0.52 g/kg) (Qiao et al., 2023). Drug dosages were calculated based on the conversions from clinical adult dosages and the body weight of rats. Rats in control and model group were smeared with blank matrix at a dose of 0.52 g/kg daily for 6 weeks. At the end of the experiment, the rats’ thyroid was examined and measured by ultrasound. After the test, all rats were fasted overnight, then weighed and anesthetized by breathing isoflurane. Blood samples were obtained via abdominal aorta, the serum was separated by centrifugation (3,500 rpm for 10 min at 4°C), and then stored at -80°C. The thyroid tissues were photographed and weighed, then fixed in 4% paraformaldehyde or kept at -80°C for subsequent analysis.

### Ultrasonic examination and measurement

Ultrasound device equipped with a high-frequency hockey stick probe (Jiangsu Dawei Electronic Equipment Co., Ltd., China) was used to examine and measure thyroid tissue in rats as previously reported (Gao et al., 2023). The cervical region of the rats was shaved to facilitate optimal probe contact. Subsequently, anesthesia was induced using isoflurane. After the application of coupler, the probe was continuously transversed from the mandible down the long axis of the neck. At the end of the transverse sweep, the probe was turned to a direction parallel to the long axis, and successive longitudinal sweeps were performed from the middle of the neck to each side. Transverse and longitudinal images of thyroid were recorded separately, and measurement packets within the ultrasound system were employed to calculate the volumes of the left and right thyroid.

### Morphological observation and organ coefficients

The anatomical thyroid tissue (with trachea) was photographed and the thyroid morphology was observed and recorded. Subsequently, the trachea was removed and the left and right thyroid glands were separated, weighed and recorded respectively. The weight of the thyroid on the left and right sides of the same rat was added to obtain the total weight of the thyroid, and the thyroid coefficients was calculated by the formula. The formula was as follows: thyroid coefficients (mg/g) = thyroid weight (mg)/body weight (g).

### Histopathological analysis

The tissues of thyroid gland were obtained for histopathological examination. In detail, the specimens of thyroid gland tissues were fixed in 4% paraformaldehyde for 48 h. After processed in a series of graded ethanol and dimethyl benzene, the tissues were embedded in paraffin, cut into 3 µm thick sections, and then stained with hematoxylin and eosin (HE). Finally, we observed pathological changes in the tissues of thyroid gland by using HS6 automatic slice analysis system (Century Sunny Technology Co., Ltd., Beijing, China).

### Detection of serum thyroid hormones level

The concentrations of FT3 (CSB-E05076r) and FT4 (CSB-E05079r) in serum were determined using ELISA kits from Cusabio biological engineering Co., Ltd. (Wuhan, China) and sample wells were spiked with 50 µL rat serum per well. The concentrations of TSH (RA20574) in serum were determined using ELISA kits from Bioswamp biological technology Co., Ltd. (Wuhan, China) and sample wells were spiked with 40 µL rat serum per well. All hormones were detected following the manufacturer’s protocols.

### mRNA sequencing

The main process of library preparation and sequencing: Thyroid glands were rapidly frozen in liquid nitrogen and stored at -80°C. Three thyroid samples each from the control group, the model group and the JJJG-treated group with the best efficacy in the comprehensive evaluation were selected for mRNA sequencing. Total RNA was isolated from the thyroids using Trizol^®^ reagent (Magen). The purity and integrity of the extracted RNA were assessed using a Nanodrop ND-2000 spectrophotometer (Thermo Fisher Scientific, United States) by measuring the A260/A280 absorbance ratio. Additionally, RNA integrity was evaluated using an Agilent Bioanalyzer 4,150 (Agilent Technologies, CA, United States) to determine the RNA integrity number (RIN value). Only RNA samples meeting the following quality criteria were used for library construction: total RNA amount ≥1 μg, OD260/280 = 1.6–1.8, OD260/230 = 1.8–2.2, RIN ≥7.0. Paired-end (PE) sequencing libraries were prepared following the manufacturer’s instructions for the ABclonal mRNA-seq Library Prep Kit (ABclonal, China). Briefly, 1 μg of total RNA was used for mRNA purification with oligo (dT) magnetic beads. The purified mRNA was then fragmented in ABclonal First-Strand Synthesis Reaction Buffer. Subsequently, cDNA synthesis was performed using random primers and reverse transcriptase (RNase H) with the fragmented mRNA as templates. This was followed by second-strand cDNA synthesis using DNA polymerase I, RNase H, reaction buffer, and dNTPs. The double-stranded cDNA fragments were then ligated with adapter sequences to facilitate PCR amplification. The PCR products were purified, and library quality was assessed using an Agilent Bioanalyzer 4,150. Finally, the libraries were sequenced on an Illumina NovaSeq 6,000 sequencing platform with a paired-end read length of 150 base pairs (bp). The resulting sequencing data generated on the Illumina platform were used for bioinformatic analysis. All RNA isolation and library construction steps were performed by Shanghai Zhongke Xinsheng Life Biotechnology Co., Ltd.

The process of bioinformatic analysis: Following generation on the Illumina platform, RNA sequencing data underwent quality control procedures. The raw sequencing data in FASTQ format were first processed using Perl scripts to remove adapter sequences and eliminate low-quality reads. Low-quality reads were defined as those containing a base quality value of ≤25 in more than 60% of the read sequence or those with an N (indicating unknown base) ratio exceeding 5%. This filtering process yielded high-quality, clean reads for subsequent analyses. HISAT2 software (http://daehwankimlab.Github.io/hisat2/) was employed to align the clean reads to a reference genome, generating mapped reads for further analysis. FeatureCounts (http://subread.sourceforge.net/) was then used to quantify the number of reads aligning to each gene. Gene expression levels were subsequently normalized using Fragments Per Kilobase Million (FPKM) values, which account for gene length. Differential expression analysis between groups was performed using DESeq2 software (http://Bioconductor.org/packages/release/bioc/html/DESeq2.html/), with a significance threshold set at |log2 fold change (FC)| > 1 and adjusted *p*-value (*P*adj) < 0.05 for identifying differentially expressed genes (DEGs). To elucidate biological pathways that may be involved following JJJG intervention in thyroid nodules rats, the DEGs were subjected to Gene Ontology (GO) and Kyoto Encyclopedia of Genes and Genomes (KEGG) enrichment analysis using the clusterProfiler R package.

### Quantitative real-time PCR(qRT-PCR)

Total RNA was extracted from the tissues of thyroid gland with Trizol (Transgen Biotech Co., Ltd., Beijing, China) following the manufacturer’s protocol. The cDNA was synthesized from 1 µg of total RNA using the reverse transcription reagent kit (Vazyme Biotech Co., Ltd., Nanjing, China) in accordance with instruction manual. The cDNA was analyzed by qRT-PCR using ChamQ SYBR qPCR Master Mix (Vazyme Biotech Co., Ltd., Nanjing, China). The sequences of the primers are listed in Table 2. The detailed composition of the qRT-PCR reaction system is listed in Table 3. The following thermocycling protocol was used: at 95°C for 30 s, 40 cycles at 95°C for 10 s and at 60°C for 30 s. The relative quantities of the candidate genes and GAPDH mRNA were calculated by comparative CT method.

**TABLE 2:** Sequences of primers used for qRT-PCR.

Genes	Forward primer (5’-3’)	Reverse primer (5’-3’)
GAPDH	GAAGGTOGOTGTGAACGCAT	CCC​ATT​TGA​TGT​TAG​CGG​GAT
IL-1β	GTG​CTG​TCT​GAC​CCA​TGT​GA	GAT​TCT​TCC​CCT​TGA​GGC​CC
IL-6	CAT​TCT​GTC​TCG​AGC​CCA​CC	GCA​ACT​GGC​TGG​AAG​TCT​CT
TNF-α	CTC​AGA​GCC​CCC​AAT​CTG​TG	TCC​AGT​GAG​TTC​CGA​AAG​CC
JAK2	AGC​CTT​GTC​ATT​TGT​GTC​GTT​AAT	ACG​GCA​AAG​GTC​AGG​AAG​TAT​TT
STAT3	TGT​CAG​ATC​ACA​TGG​GCT​AAG​TT	TGA​AAC​CCA​TGA​TGT​ACC​CTT​CA
VEGF	GGC​CTC​TGA​AAC​CAT​GAA​CTT​TC	ACA​CAG​GAC​GGC​TTG​AAG​ATA​TA

**TABLE 3:** The detailed composition of the qRT-PCR reaction system.

Reagent name	Addition volume (µL)
2×ChamQ SYBR qPCR Master Mix	10
Forward primer	0.4
Reverse primer	0.4
50×ROX Reference Dye 2	0.4
Template DNA	2
ddH_2_O	6.8

### Detection of IL-1β, IL-6 and TNF-α protein content

The protein content of IL-1β, IL-6 and TNF-α were determined using ELISA kits. The thyroid gland tissues from each group were cut into pieces and homogenized in phosphate-buffered saline (PBS) buffer (pH = 7.4) to extract total protein. The supernatant was collected following centrifugation at 4,000 rpm for 20 min at 4°C, and was determined according to the manufacturer’s protocols (Cusabio biological engineering Co., Ltd., Wuhan, China).

### Western blotting analysis

Thyroid tissues were homogenized in radioimmunoprecipitation assay (RIPA) buffer containing 1% phenylmethylsulfonyl fluoride (PMSF) and 1% phosphatase inhibitor cocktail. Total protein was extracted using a protein extraction kit following the manufacturer’s instructions (Bioss, Wuhan, China). Total protein was quantified by the BCA protein assay reagent (Boster Bio-engineering Co., Ltd., Wuhan, China), separated by 10% sodium dodecyl sulfate polyacrylamide gel electrophoresis (SDS-PAGE), and transferred to polyvinylidene fluoride (PVDF) membranes. 5% non-fat milk (milk powder dissolved in TBS supplemented with 0.1% Tween 20) was used to block the PVDF membrane. Then, the membranes were incubated with primary antibodies configured in the blocking solution (JAK2, STAT3, p-STAT3 (Tyr705), VEGF and β-actin, all at 1:1,000 dilutions; p-JAK2 at 1:800 dilutions) overnight at 4°C. The membranes were washed 5 times with TBST (TBS supplemented with 0.1% Tween 20) for 5 min each time. Subsequently, the membranes were incubated with secondary antibodies (Bioss, bs-80295G-HRP) configured in the blocking solution for 1 h at room temperature. The membranes were washed 5 times with TBST for 5 min each time. The bands were visualized using a chemiluminescence reagent (EMD Millipore Corporation, WBKLS0100, Burlington, MA, United States). Imaging was performed using 4800Multi automatic image analysis system (Tanon technology Co., Ltd., Shanghai, China). Protein bands were quantified using ImageJ software with β-actin as the internal control. Antibodies to JAK2 (Bioss, bs-0908R), STAT3 (Bioss, bs-1141R), p-JAK2 (ABclonal, AP0531), p-STAT3 (Tyr705) (Bioss, bs-1658R), VEGF (Bioss, bs-20393R), and β-actin (Bioss, bs-0061R) were used in this study.

### Immunofluorescence staining

The sections were dewaxed to water, and the tissue was incubated with 3% hydrogen peroxide for 10 min. The antigens were repaired after the tissues were microwaved for 16 min with 1×sodium citrate antigen retrieval solution (pH = 6.0, Bioss, C02-02002). The sections were blocked with 10% goat serum (Bioss, C01-03001), and then incubated with anti-VEGF antibody diluted 1:300 with antibody diluent (Bioss, C01-04001) at 4°C overnight. The sections were washed 4 times with PBST (PBS supplemented with 0.1% Tween 20) for 5 min each time. Subsequently, the specimens were incubated with secondary antibodies (a Cy3-conjugated goat anti-rabbit secondary antibody) diluted 1:500 with fluorescent antibody dilution buffer (Bioss, C01-04004) for 1 h at room temperature. The sections were washed 4 times with PBST for 5 min each time. The nuclei were stained with DAPI solution (Bioss, C02-04002) for 5 min, and the sections were sealed with antifading mounting medium (Solarbio, S2100). Images were obtained at 555 nm excitation wavelength and 570 nm emission wavelength using Axio Imager.A2 advanced fluorescence microscope (Olympus Corporation, Tokyo, Japan) with Zen fluorescence analysis software.

### Statistical analysis

Statistical analysis was performed with Graphad Prism 8.02 software. All data were presented as mean ± SE. Differences in mean values of various groups were analyzed by one-way analysis of variance (ANOVA). Comparisons of numerical data between two groups were calculated by least significant difference (LSD) tests. Difference with *P*-value <0.05 was considered as statistically significant.

## Results

### Quantitative analysis of effective components in JJJG

**FIGURE 2:**
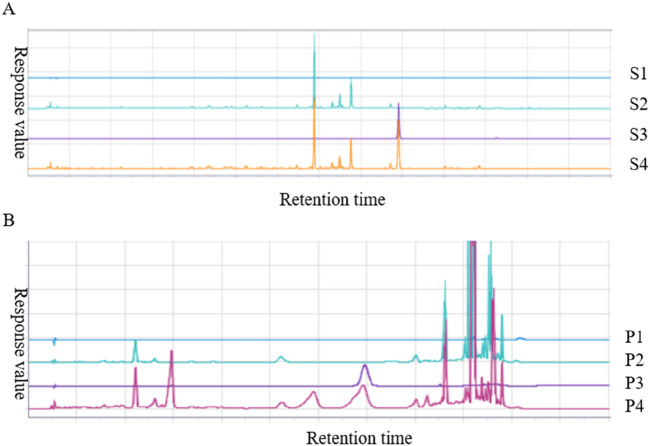
HPLC analysis chromatogram of paeonol **(A)** and forsythoside A **(B)** in JJJG. S1-S4 represented blank solvent, negative gel sample of *Moutan Cortex*, paeonol standard and test sample of JJJG, respectively. P1-P4 represented blank solvent, negative gel sample of *Forsythiae Fructus*, forsythoside A standard and test sample of JJJG, respectively.

Paeonol is the most important active component in *Moutan Cortex* (Lv et al., 2020), which can inhibit the proliferation of thyroid cancer cells by affecting the lnc RNA expression (Xiang et al., 2015). Forsythoside A is the main pharmacological active ingredient in *Forsythiae Fructus*. Through network pharmacological data analysis and experimental studies, it was considered that forsythoside A was one of the active ingredients in the treatment of goiter (Li, 2017b). Therefore, paeonol and forsythoside A were selected as the main pharmacodynamic components of JJJG to determine their content. The characteristic chromatograms of the standards, negative gel samples and JJJG were shown in Figure 2. We adopted related chemical standards to quantify levels of paeonol and forsythoside A, respectively. To be specific, the contents of the paeonol and forsythoside A were 1.160 and 0.608 mg/g in JJJG, respectively (Table 4).

**TABLE 4:** Contents of paeonol and forsythoside A in JJJG.

Sample name	Paeonol (mg/g)	Forsythoside A (mg/g)
1	1.153	0.613
2	1.164	0.604
3	1.162	0.607
Average	1.160	0.608

### JJJG improved nodular lesions and reduced thyroid volume

**FIGURE 3:**
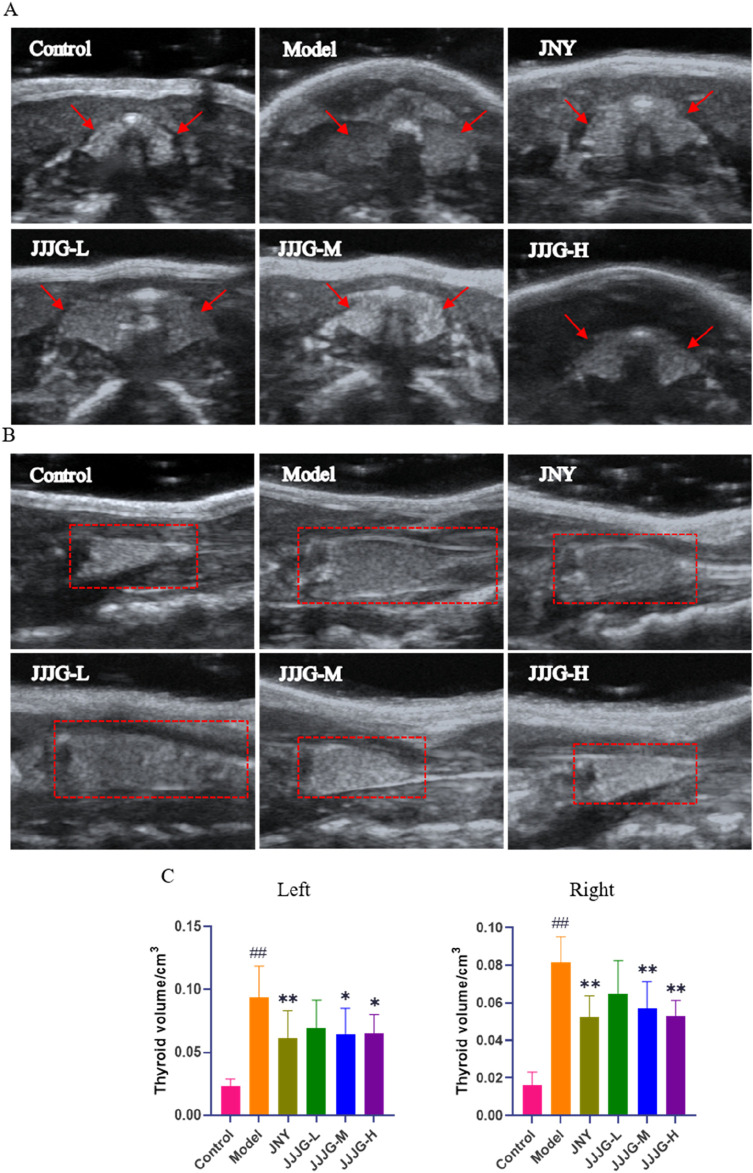
JJJG improved nodular lesions and reduced thyroid volume. **(A)** Thyroid image during ultrasonic cross-section scan. **(B)** Thyroid image during longitudinal ultrasound scan. **(C)** The volume of thyroid on the left and right sides of rats. Data were shown as mean ± SE (n = 10), one-way ANOVA followed by LSD tests were used for comparison between groups, ^##^ *P* < 0.01 vs. control group; ^*^ *P* < 0.05 and ^**^ *P* < 0.01 vs. model group.

Ultrasonography has high sensitivity, specificity and safety, and is currently the gold standard in clinical diagnosis of thyroid nodules (Chu et al., 2021). We used ultrasonography to examine and measure thyroid tissue in rats to analyze the construction of thyroid nodules model and evaluate the therapeutic effect of JJJG. Ultrasound images showed that the transverse section of the thyroid was butterfly shaped, and the longitudinal section was a triangle with a narrow top and wide bottom. In control group, the echo of thyroid parenchyma was uniform, the boundary was clear, and the anteroposterior diameter, left and right diameter and upper and lower diameter were smaller. In model group, the glands on both sides showed obvious asymmetry enlargement and the boundary was blurred and irregular. There are strong echoes of punctural or striated fibrous hyperplasia caused by nodular lesions. The comparison of ultrasound images between the control group and model group confirmed the successful establishment of the thyroid nodules rat model. The JNY, JJJG-M and -H group showed obvious therapeutic effect. The anteroposterior diameter, left and right diameter and upper and lower diameter were significantly reduced, the boundary was clear and relatively regular, the echo was relatively uniform, and the nodule lesions were significantly improved (Figures 3A, B). The thyroid volume on both sides of model rats were markedly increased by PTU administration compared with control group (*P* < 0.01). After 6 weeks’ treatment, the thyroid volume on both sides of the JNY, JJJG-M and -H group were obviously decreased (*P* < 0.05), while there was no significant difference in JJJG-L group (*P* > 0.05). The therapeutic effect of JJJG was dose-dependent. The change in thyroid volume is shown in Figure 3C.

### JJJG reduced thyroid swelling and organ coefficients

**FIGURE 4:**
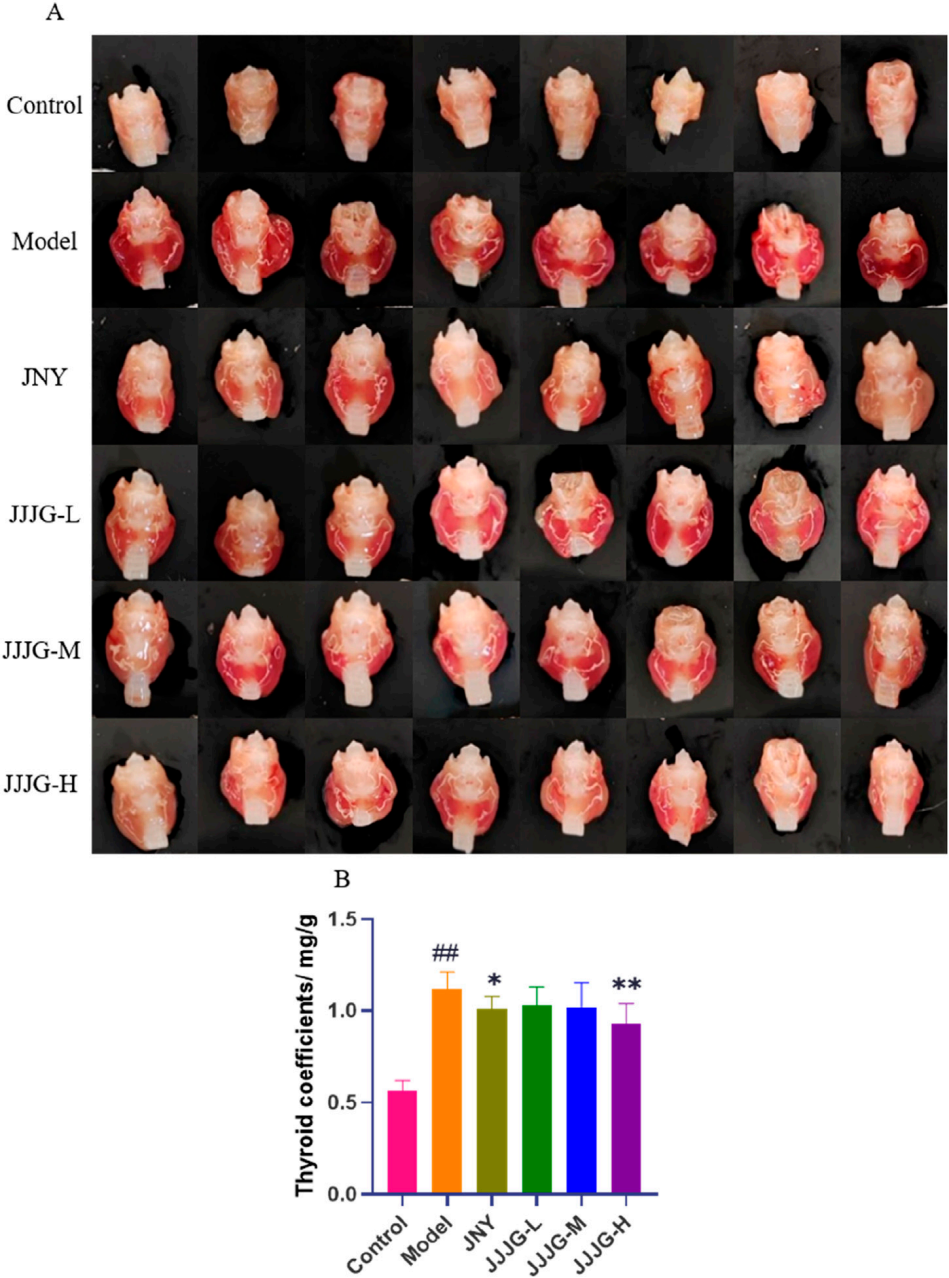
JJJG reduced thyroid swelling and organ coefficients. **(A)** Representative pictures of thyroid morphology in rats. **(B)** Organ coefficients of thyroid in rats. Data were shown as mean ± SE (n = 14), one-way ANOVA followed by LSD tests were used for comparison between groups, ^##^ *P* < 0.01 vs. control group; ^*^ *P* < 0.05 and ^**^ *P* < 0.01 vs. model group.

To further investigate the effects of JJJG on thyroid nodules, morphology and organ coefficients of thyroid in rats of each group were obtained. As shown in Figure 4A, the left and right lobes of the thyroid in control group were small and pink in color. On the other hand, Morphological observation from rats in the model group showed extensive pathological changes, the left and right lobes of the thyroid were swollen, the tissue was bloodshot, and the color was dark red. After JJJG and JNY treatment, the swelling of the left and right lobes of the thyroid was alleviated to varying degrees, and the tissue color was mainly light red. In addition, the thyroid coefficients were significantly increased in the model group compared with the control group (*P* < 0.01). However, it reduced significantly after the treatment with JJJG-H (*P* < 0.01) and JNY (*P* < 0.05). There were no outstanding differences between the JJJG-L, -M and model groups (*P* > 0.05; Figure 4B). To sum up, the results showed that in comparison with the control group, the thyroid of the model group was seriously swollen and the organ coefficients increased significantly, suggesting that the model was successfully established. In contrast, the swelling and increased organ coefficients induced by PTU recovered significantly in the rats treated with JJJG.

### JJJG attenuated nodular hyperplasia of epithelial cells in thyroid tissue

**FIGURE 5:**
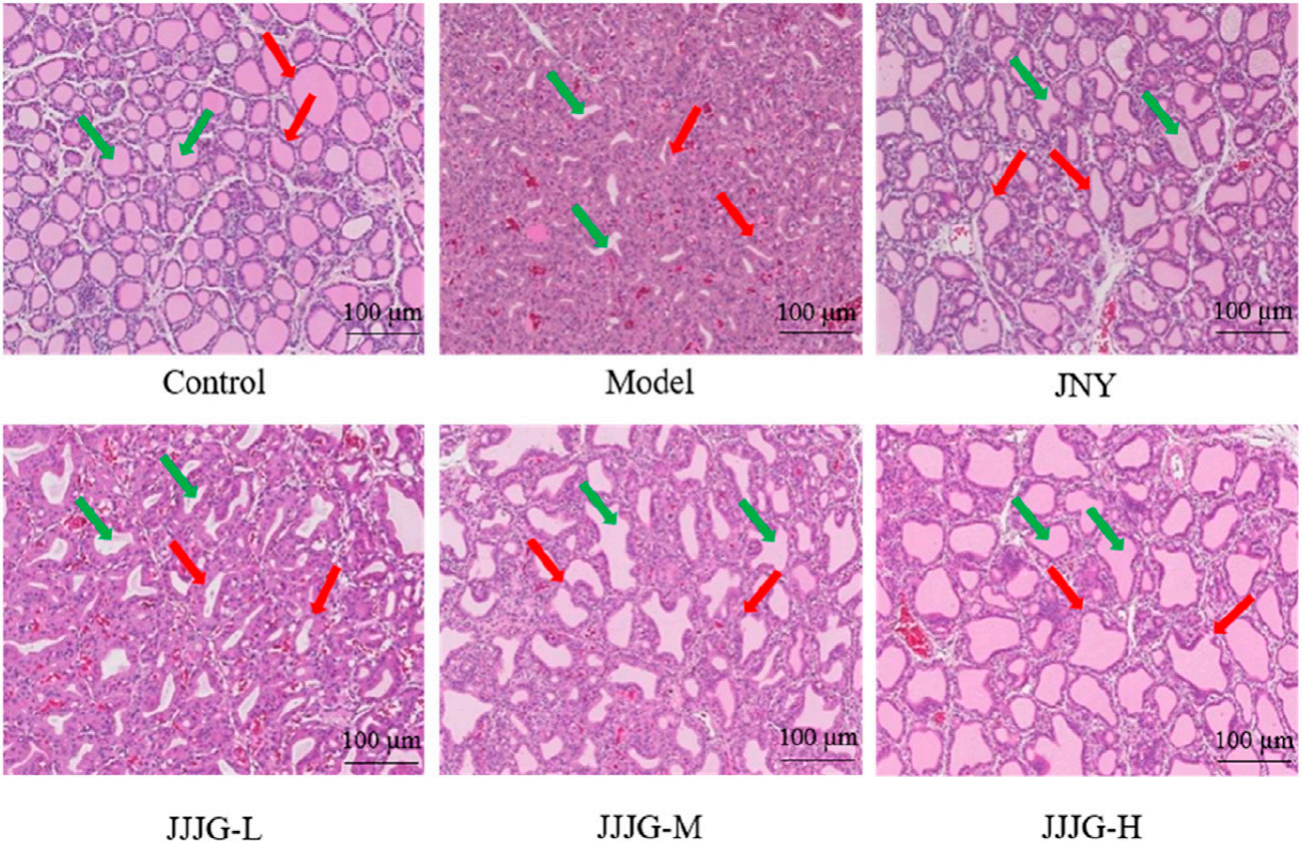
The pathological changes of thyroid tissue in rats were observed by HE staining (original magnification, 200×). The green arrow marked the thyroid follicles and the red arrow marked the thyroid follicular epithelial cells.

To verify the effectiveness of JJJG in mitigating thyroid nodules, HE staining was conducted and histological variation was observed. As can be seen from Figure 5, there were a large number of round or oval follicles in the thyroid of the control group rats, which were medium in size and regular in shape, and the epithelial cells were cubic or flat in regular arrangement, and there was no abnormal nodular hyperplasia in loose connective tissue between cells and follicles. In contrast in the model group, the follicles in the thyroid were few and irregularly arranged, the epithelial cells were highly nodular hyperplasia, and the boundaries between the follicles were blurred, which indicated the success of the model preparation. Administration of JNY and JJJG-H dose significantly suppressed these typical histological patterns. The shape and arrangement of the follicles were relatively regular, and the size was relatively uniform. There was almost no abnormal nodular hyperplasia in the loose connective tissue between cells and follicles, the boundaries between follicles were relatively clear, and the thyroid tissue structure basically returned to normal. While daily treatment of JJJG-L and -M dose for 6 weeks, compared to the model group, were also capable to alleviate follicular epithelial nodular hyperplasia in different degree. These results illustrated that JJJG had therapeutic effect on the rats with thyroid nodules induced by PTU.

### JJJG modulated the levels of serum FT3, FT4 and TSH

**FIGURE 6:**
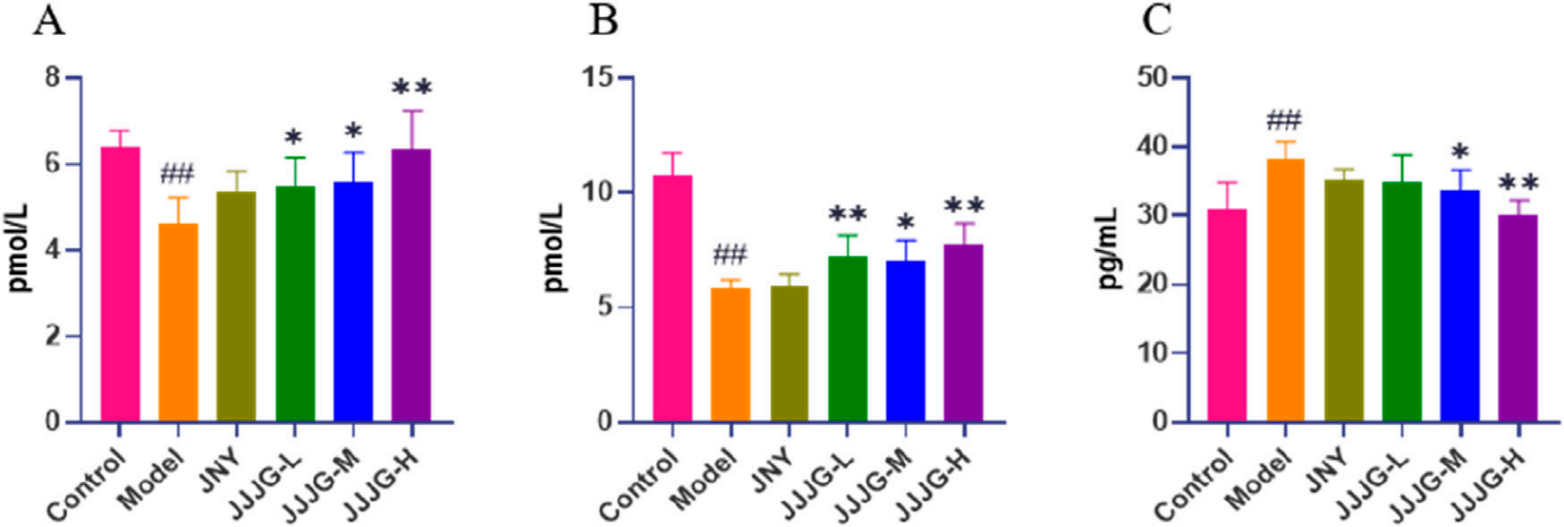
JJJG modulated serum thyroid hormones level. FT3 **(A)**, FT4 **(B)** and TSH **(C)**. Data were shown as mean ± SE (n = 10), one-way ANOVA followed by LSD tests were used for comparison between groups, ^##^ *P* < 0.01 vs. control group; ^*^ *P* < 0.05 and ^**^ *P* < 0.01 vs. model group.

Modern medicine believes that thyroid dysfunction can lead to the proliferation of thyroid epithelial cells, resulting in the formation of thyroid nodules (Zhang et al., 2019). Quantitative analysis of thyroid hormones can reveal the regulatory relationship between HPT axis and reflect the function of thyroid sensitively (Ortiga-Carvalho et al., 2016). The results showed that compared with control group, FT3 and FT4 levels were obviously decreased (*P* < 0.01 for both cases), while TSH level was notably increased in the model group (*P* < 0.01), which was consistent with the current evaluation criteria of thyroid nodules animal model (He et al., 2019; Wang et al., 2021). The administration of JJJG at medium and high doses increased the levels of FT3 and FT4 (*P* < 0.05) and decreased the level of TSH (*P* < 0.05). JJJG has a certain dose dependence in regulating three hormones levels. Notably, there were no significant differences in serum FT3, FT4 and TSH levels between JNY and model group (*P* > 0.05). Our results suggested that JJJG could adjust the serum FT3, FT4 and TSH levels to play anti-thyroid nodules effect (Figures 6A–C).

### Gene expression analysis by mRNA sequencing

**FIGURE 7:**
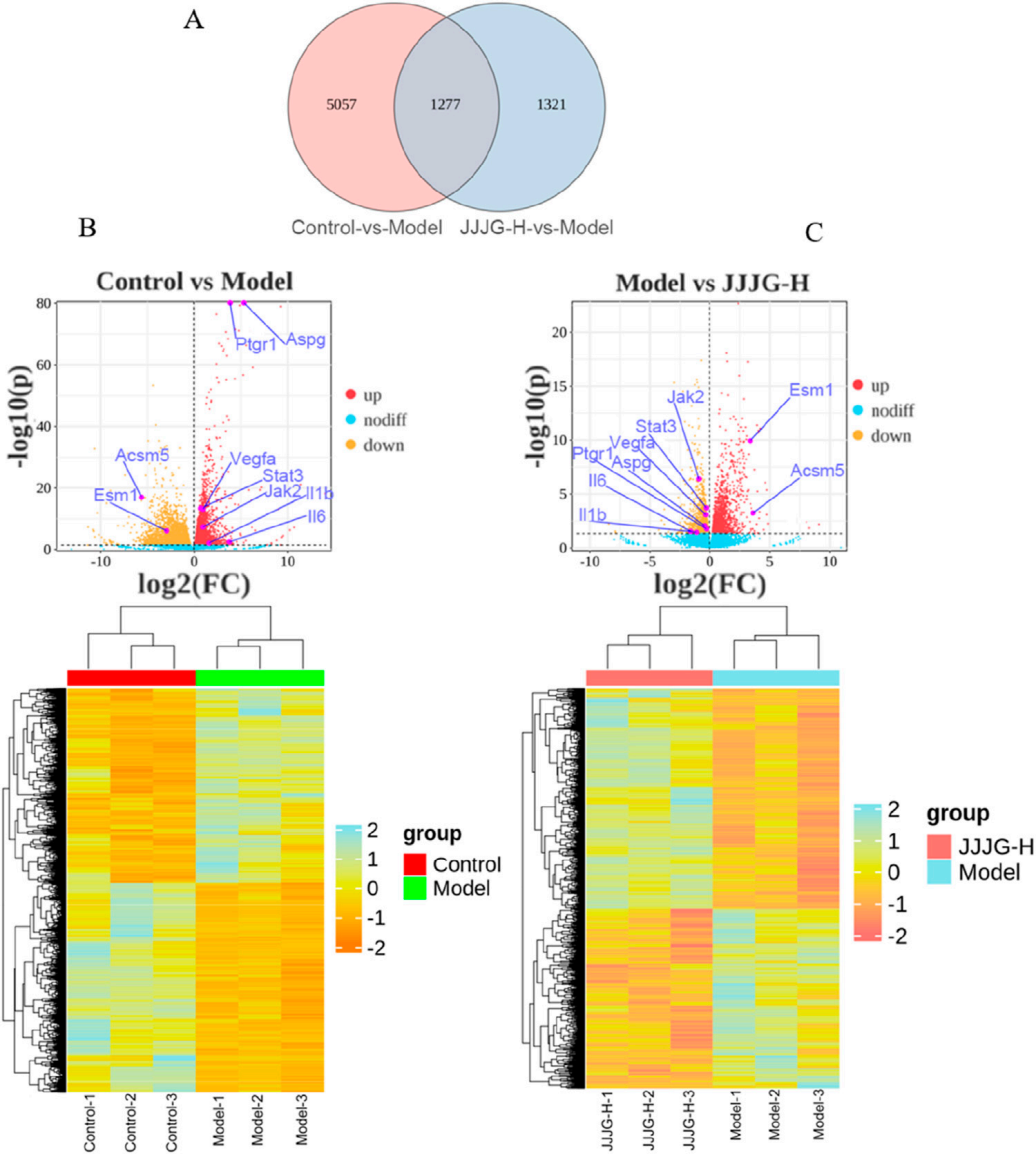
Gene expression analysis of the mRNA sequencing. **(A)** Overlapping DEGs between model vs. control and JJJG-H vs. model (Venn). **(B)** Hierarchical clustering thumbnail and DEG volcano plot between the control group and the model group. **(C)** Hierarchical clustering thumbnail and DEG volcano plot between the model group and the JJJG-H group.

According to the above pharmacodynamic comprehensive evaluation results, it can be seen that the therapeutic effect of JJJG-H group was better, so we selected the control group, model group and JJJG-H group for thyroid transcriptional analysis. DEGs were filtered based on *P*_*adj*_ < 0.05 and |log_2_(Fold Change)|>1. The overlapping DEGs between model vs. Control and JJJG-H vs. model are shown in Figure 7A. We detected 3,060 upregulated genes and 3,274 downregulated genes between the model and control groups, 1,437 upregulated genes and 1,161 downregulated genes in the comparison of JJJG-H versus the model group, respectively. Key genes involved in thyroid nodules pathogenesis and JJJG treatment were revealed, such as *Il6*, *Il1b*, *Jak2*, *Stat3* and *Vegfa*, etc. (Figures 7B, C).

### Gene ontology and kyoto encyclopedia of genes and genomes enrichment analysis on overlapping DEGs

**FIGURE 8:**
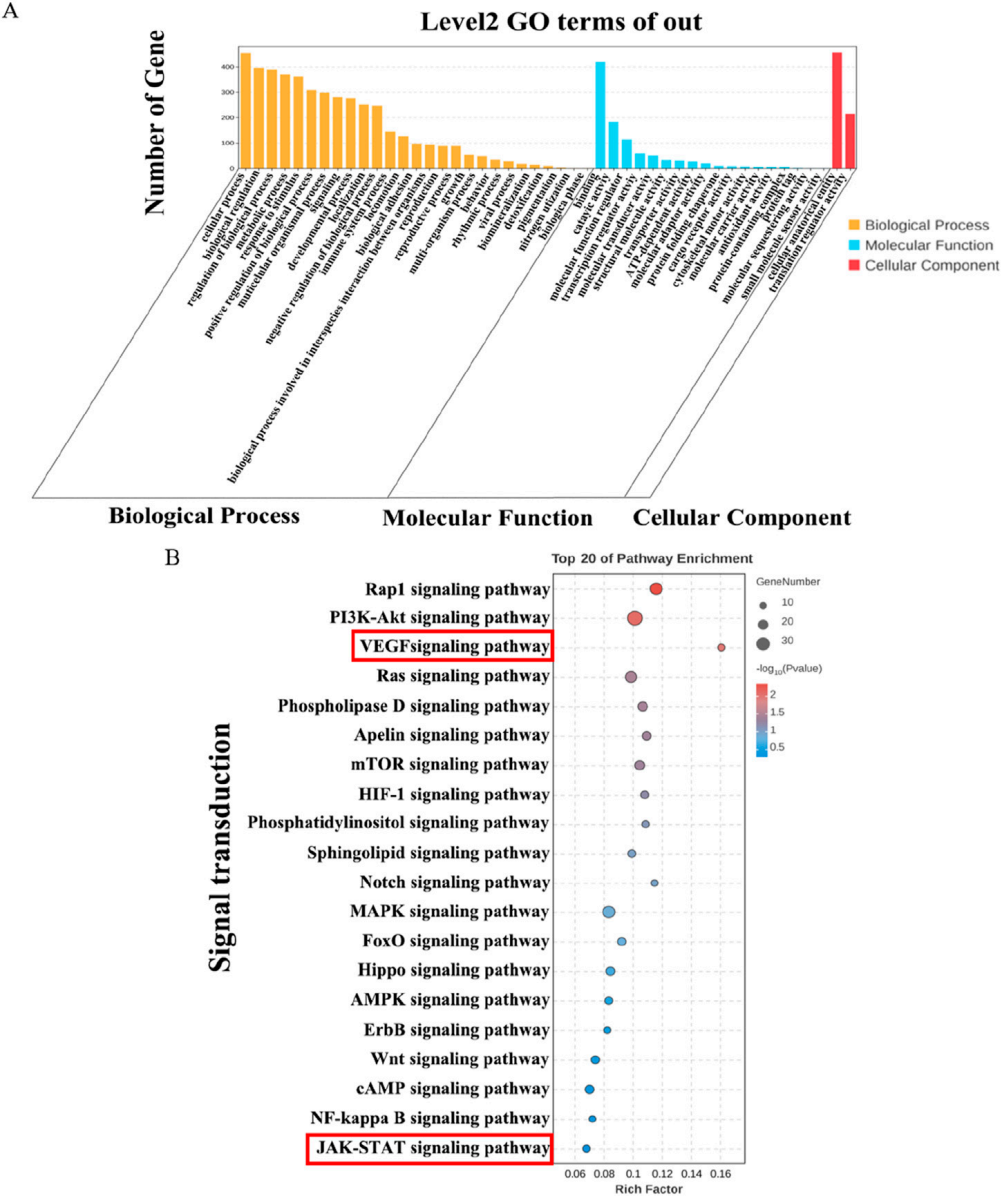
GO and KEGG enrichment analysis on overlapping DEGs. **(A)** GO analysis histogram of the 1,277 overlapping DEGs between model vs. control and JJJG-H vs. model. **(B)** KEGG enrichment analysis of the 1,277 overlapping DEGs between model vs. control and JJJG-H vs. model.

To clarify the functions and signaling cascades enriched upon JJJG treatment in PTU-induced thyroid nodules, 1,277 overlapping DEGs between model vs. control and JJJG-H vs. model were used for GO and KEGG enrichment analysis. GO analysis indicated that DEG targets of JJJG participated in the regulation of biological process, metabolic process, and immune system process. The molecular functions involved mainly include molecular transducer activity and antioxidant activity, etc (Figure 8A). KEGG analysis suggested that the overlapping DEGs were closely related to signal transduction pathways including rhoptry-associated protein 1(Rap1), PI3K-AKT, VEGF and JAK-STAT (Figure 8B).

Bulk RNA sequencing analysis showed the increased expression of proinflammatory factors (*Il6* and *Il1b*), *Jak2*, *Stat3* and *Vegfa* after PTU gavage, along with its decrease upon JJJG treatment (Figures 7B, C). In KEGG analysis, we observed the significant enrichment of VEGF and JAK-STAT signaling pathways in the overlapping DEGs between model vs. control and JJJG-H vs. model (Figure 8B). Furthermore, relevant scholar has demonstrated proinflammatory factors (e.g., IL-6, TNF-α, IL-1β) can affect angiogenesis via the JAK2/STAT3/VEGF signaling pathway (Cheng et al., 2020). Increased angiogenesis was thought to be one of the key mechanisms in the formation of thyroid nodules (Haytaoglu et al., 2019; Viacava et al., 2007), and VEGF promoted vascular endothelial cell proliferation and neovascularisation (Apte et al., 2019). JAK2/STAT3 pathway also played a key role in driving angiogenesis and inflammatory crosstalk in papillary thyroid cancer cells (Zhao et al., 2021; Pan et al., 2019), but there were no reports on this signaling pathway in the pathogenesis of thyroid nodules at present. Besides, accumulating evidences have demonstrated that paeonol and forsythoside A, the effective components in JJJG, can act on the key pathological mechanism of (IL-6, TNF-α, IL-1β)/JAK2/STAT3/VEGF in the treatment of liver cancer and acute lung injury (Yu and Liang, 2017; Lu et al., 2020). Therefore, through transcriptomics in conjunction with literatures, we speculated that JJJG may treat thyroid nodules via (IL-6, TNF-α, IL-1β)/JAK2/STAT3/VEGF pathway. Notably, this pathway was also explored for the first time in thyroid nodules.

### JJJG inhibited the mRNA expression of IL-6, TNF-α, IL-1β, JAK2, STAT3 and VEGF

**FIGURE 9:**
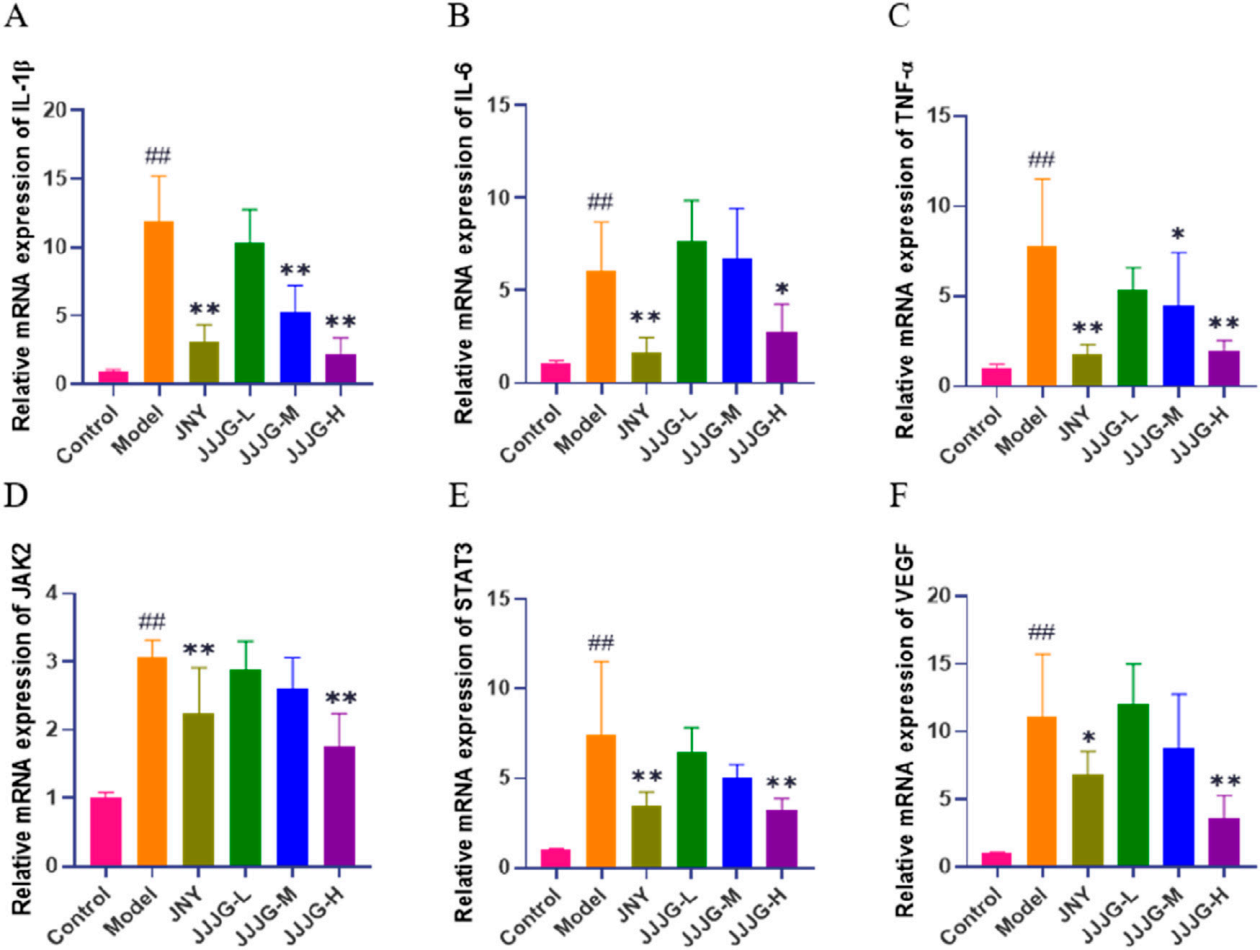
JJJG inhibited mRNA expression level of key factors. IL-1β **(A)**, IL-6 **(B)**, TNF-α **(C)**, JAK2 **(D)**, STAT3 **(E)** and VEGF **(F)**. Data were shown as mean ± SE (n = 3), one-way ANOVA followed by LSD tests were used for comparison between groups, ^##^ *P* < 0.01 vs. control group; ^*^ *P* < 0.05 and ^**^ *P* < 0.01 vs. model group.

To validate our results, we detected the expression of relevant genes associated in the (IL-6, TNF-α, IL-1β)/JAK2/STAT3/VEGF signaling pathway using qRT-PCR. The results showed that the mRNA expression levels of IL-1β, IL-6, TNF-α, JAK2, STAT3 and VEGF in thyroid tissue of model rats were significantly higher than those of control group (*P* < 0.01). Conversely, treatment with JNY and high-dose JJJG inhibited the mRNA expression of IL-1β, IL-6, TNF-α, JAK2, STAT3 and VEGF (*P* < 0.05). In addition, the mRNA expression of IL-1β and TNF-α were reduced in JJJG-M group rats compared with that in model rats (*P* < 0.05). In summary, JJJG was able to reduce mRNA expression levels of the above six key factors in a dose-dependent manner, which was consistent with the results of mRNA sequencing. (Figures 9A–F).

### JJJG reduced the protein content of IL-1β, IL-6 and TNF-α

**FIGURE 10:**
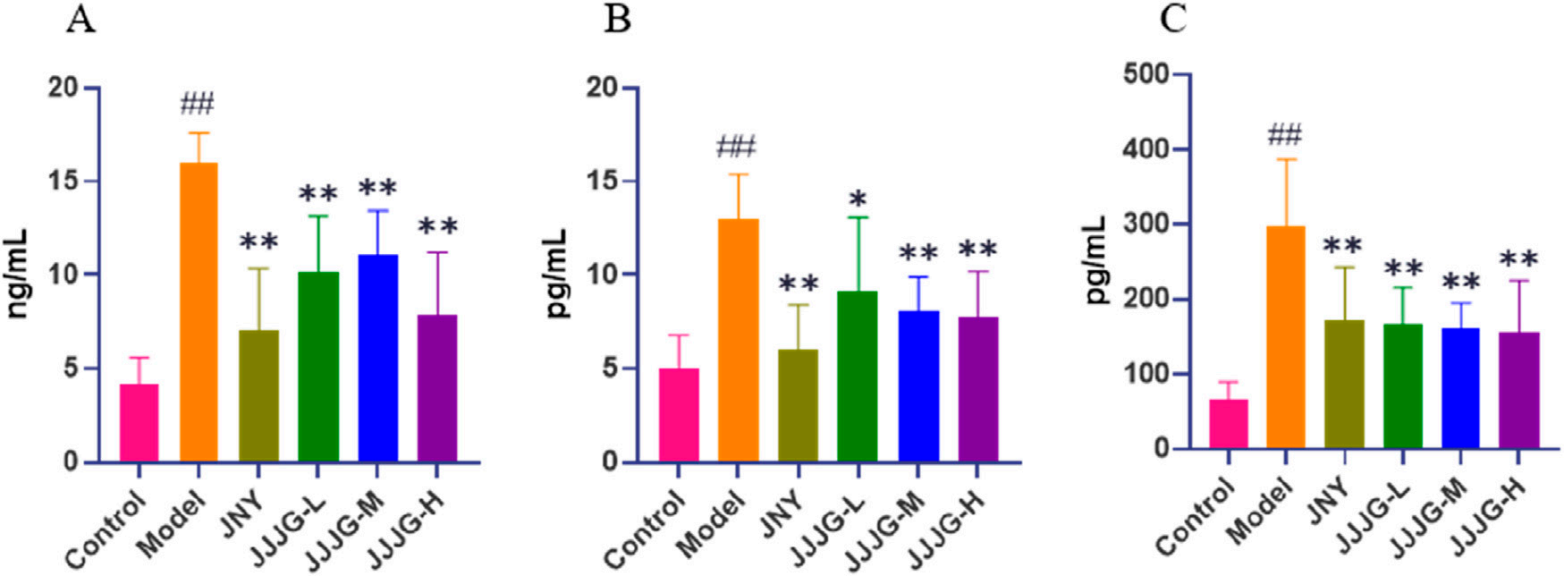
JJJG reduced the protein content of inflammatory cytokines. IL-1β **(A)**, IL-6 **(B)** and TNF-α **(C)**. Data were shown as mean ± SE (n = 8), one-way ANOVA followed by LSD tests were used for comparison between groups, ^##^ *P* < 0.01 vs. control group; ^*^ *P* < 0.05 and ^**^ *P* < 0.01 vs. model group.

At the protein plane, we first used ELISA to detect the protein content of three inflammatory cytokines in thyroid tissue. Tests for inflammatory cytokines showed significantly higher protein contents of IL-1β, IL-6 and TNF-α in the model group than those in the control group (*P* < 0.01). The protein contents of the above indicators significantly decreased after the treatment of JNY and JJJG compared with the model group (*P* < 0.05; Figures 10A–C).

### JJJG downregulated the protein expression of JAK2/STAT3/VEGF pathway

**FIGURE 11:**
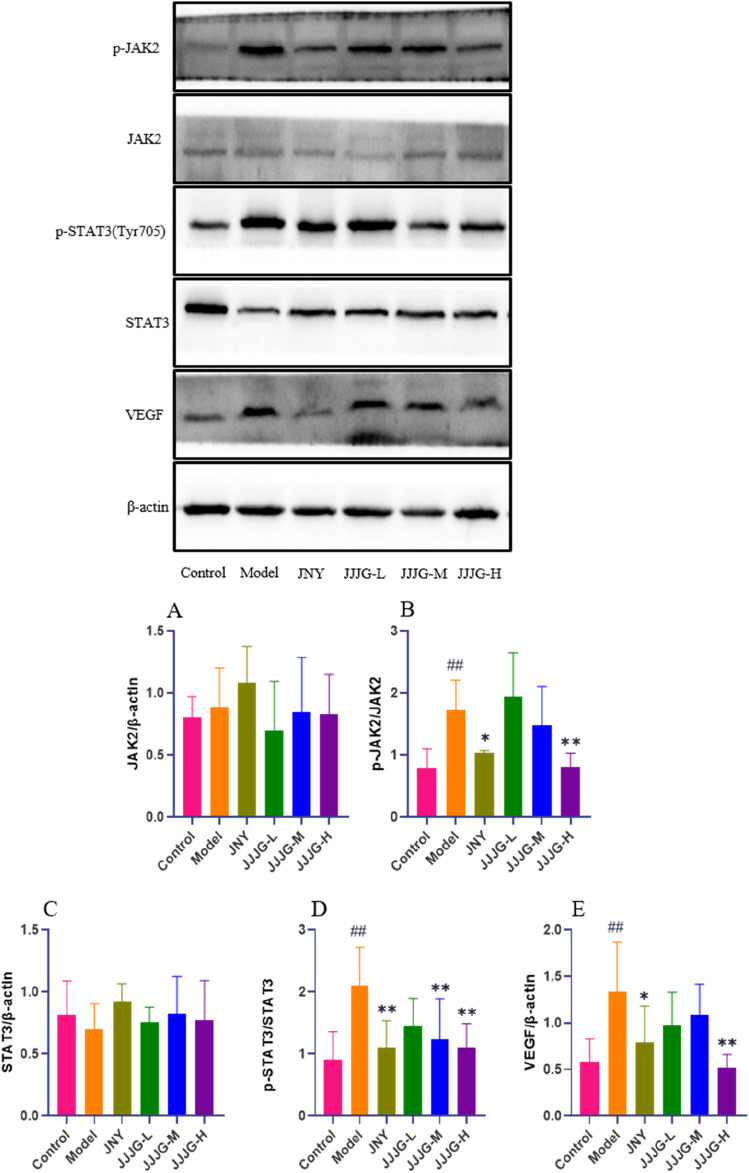
JJJG downregulated the protein expression of JAK2/STAT3/VEGF pathway. The expression levels of JAK2/β-actin **(A)**, p-JAK2/JAK2 **(B)**, STAT3/β-actin **(C)**, p-STAT3 (Tyr705)/STAT3 **(D)** and VEGF/β-actin **(E)** were assessed by western blotting. Data were shown as mean ± SE (n = 3), one-way ANOVA followed by LSD tests were used for comparison between groups, ^##^ *P* < 0.01 vs. control group; ^*^ *P* < 0.05 and ^**^ *P* < 0.01 vs. model group.

Next, western blotting was performed to determine protein expression in the JAK2/STAT3/VEGF signaling pathway. As shown in Figure 11, we found that the protein expression levels of p-JAK2/JAK2, p-STAT3 (Tyr705)/STAT3 and VEGF were significantly higher in the model group than those in the control group (*P* < 0.01). However, the expression levels of the above proteins were significantly reduced (*P* < 0.05) after JJJG-H and JNY interventions. There were no significant differences in the protein expression levels of JAK2 and STAT3 in thyroid tissues among all groups (*P* > 0.05). Consequently, as further confirmed by ELISA and WB assays (Figures 10, 11), JJJG significantly inhibited the (IL-6, TNF-α, IL-1β)/JAK2/STAT3/VEGF signaling pathway.

### JJJG inhibited VEGF expression in follicular epithelial cells of thyroid tissue

**FIGURE 12:**
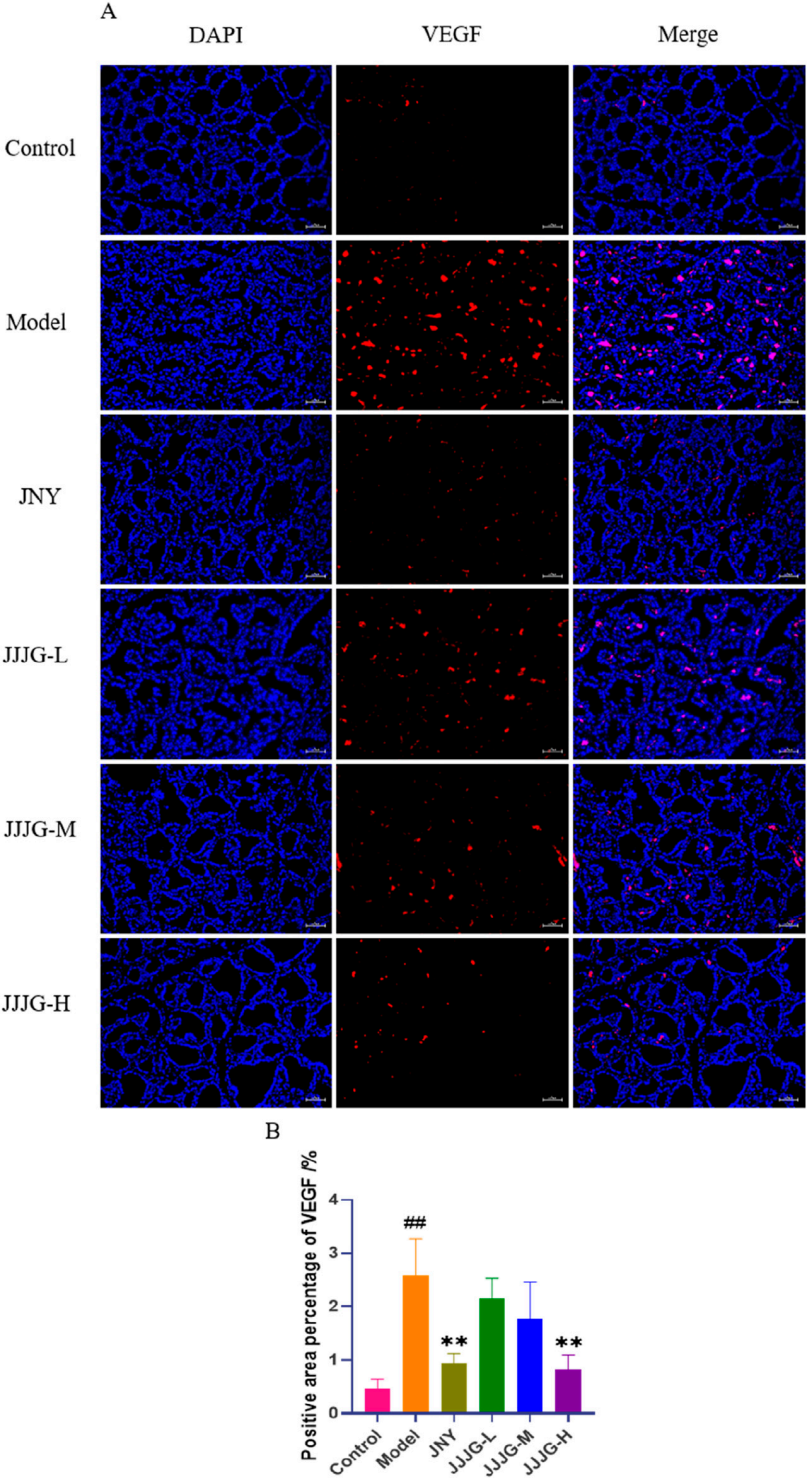
JJJG inhibited VEGF expression in follicular epithelial cells of thyroid tissue. **(A)** Immunofluorescence staining for VEGF (red) and DAPI (blue) in rat thyroid (original magnification, 200×). **(B)** The positive area percentage of VEGF in thyroid. Scale bar = 50 µm. Data were shown as mean ± SE (n = 3), one-way ANOVA followed by LSD tests were used for comparison between groups, ^##^ *P* < 0.01 vs. control group; ^**^ *P* < 0.01 vs. model group.

The role of angiogenesis in the progression of thyroid nodules has received important attention (Fu et al., 2021). Related study has found a significant increase in angiogenesis in swollen thyroid tissue (Bidey et al., 1999). VEGF is not only an important factor in regulating angiogenesis, but also the ultimate foothold of the mechanism exploration. Therefore, immunofluorescence staining was used to examine the location and expression of VEGF proteins in paraffin sections of thyroid tissues. The positive expression of VEGF in the model group was significantly higher than that in the control group (*P* < 0.01), and predominantly in follicular epithelial cells, indicating a significant increase in angiogenesis. Compared with the model group, all the medication groups reduced the expression of VEGF in thyroid follicular epithelial cells and neovascularisation to varying degrees, especially the JNY group and the JJJG-H group (*P* < 0.01; Figures 12A, B). Therefore, consistent with the WB assay, JJJG ameliorated thyroid nodules by inhibiting VEGF expression.

## Discussion

### JJJG had a good therapeutic effect on thyroid nodules

Histopathological analysis and organ coefficients determination are the main pharmacodynamic evaluation methods in the current thyroid nodule animal experiments (Ge et al., 2021; Qi and Gao, 2012). Ultrasound imaging technology is an authoritative method for the diagnosis of thyroid nodules, which is widely used in clinical practice, but rarely used in laboratory studies (Cao et al., 2023; Wang et al., 2023b). Ultrasonography enables living detection and avoids the irreversible defects caused by the execution of rats for detection, and has the advantages of high efficiency, convenience, safety and non-invasiveness, which can be promoted and applied in future animal experimental studies of thyroid nodules. In this study, while applying ultrasonography, we evaluated the efficacy of JJJG by using comprehensive means such as morphological observation, organ coefficients determination and histopathological analysis, all of which showed that JJJG had a good therapeutic effect on thyroid nodules, which was able to reduce swelling, inhibit hyperplasia, and ameliorate the nodular lesions of the thyroid tissues.

Regarding the selection of positive control drugs, since JJJG is an external preparation, to ensure the same administration mode, oral ones such as euthyrox and Xiakucao capsules, which are often used as positive control drugs, were not suitable in this study. However, there has not yet been an external preparation with a national drug approval number for the treatment of thyroid nodules. In order to ensure the reliability and authority of the positive control drug, JNY, an external preparation for the treatment of thyroid nodules that has been approved by the State Drug Administration under the medical device code and has been marketed and sold, was selected.

In addition, patients with thyroid nodules are also prone to depression and other adverse emotions due to limitations in their understanding of the disease. Yin et al. found a high co-morbidity probability between thyroid nodules and depressed mood by measuring depression levels in 1,688 medical examiners (Yin et al., 2019). Another clinical study used a depression self-assessment scale to score 120 patients with thyroid nodules, which showed a score of 49.96, indicating that patients with thyroid nodules developed significant depression (Yu et al., 2023a). Relevant studies have proved that peppermint oil and *Borneolum* can have a good antidepressant effect (Yu et al., 2023b; Wang et al., 2019). By adding peppermint oil and *Borneolum* in JJJG, it facilitates the transdermal absorption of the drug while also favouring the improvement of depression (Li et al., 2023).

### Analysis of the therapeutic mechanism of JJJG on thyroid nodules

Thyroid nodules may occur due to insufficient thyroid hormone synthesis and negative feedback regulation that causes the pituitary gland to secrete more TSH, which increases the stimulation of the thyroid tissues and causes them to proliferate and form nodules (Suzuki et al., 2022). Therefore, it is essential to observe the effect of drugs on the regulation of thyroid hormones levels. The functional status of the thyroid in clinical practice is often reflected by the levels of FT3 and FT4, combined with TSH (Zuo et al., 2010). Quantitative results showed that JJJG could significantly increase FT3 and FT4 and decrease TSH levels, thus reducing the stimulation on thyroid follicular epithelial cells and inhibiting hyperplasia. It has been reported that Huoxue Xiaoying recipe can also increase the serum FT3 and FT4 and decrease the TSH content in the model rats with thyroid nodules (Pei et al., 2022). The effects of both in the regulation of thyroid hormones levels are consistent. In contrast, the positive control drug JNY had no significant effect in regulating serum levels of FT3, FT4 and TSH, suggesting that JJJG may have richer action routes and targets than JNY.

mRNA sequencing is a powerful, high-throughput sequencing technique that offers distinct advantages for studying gene expression in thyroid nodules. Unlike traditional methods, mRNA sequencing enables comprehensive detection of the complete transcriptome, encompassing both known and unknown genes. Additionally, mRNA sequencing boasts high resolution and accuracy, allowing for the detection of lowly expressed genes and the precise quantification of highly expressed genes within the thyroid nodule transcriptome. This powerful technique has the potential to reveal novel gene functions and signaling pathways, thus providing a new thinking about disease pathogenesis and treatment, and helping to develop more therapeutic strategies. The results suggested that the pathogenesis and therapeutic mechanism may be associated with altered expression of specific genes, including *Il6*, *Il1b*, *Jak2*, *Stat3* and *Vegfa*, etc. Furthermore, it is noteworthy that our research not only explored the well-established PI3K-Akt and Ras signaling pathways implicated in thyroid nodules but also shed light on lesser-studied pathways in this context, such as the (IL-6, TNF-α, IL-1β)/JAK2/STAT3/VEGF signaling pathway, which contributes to a more comprehensive and in-depth understanding of thyroid nodules and provides a basis for the development of new drugs.

Inflammatory factors (IL-6, TNF-α, IL-1β, etc.) can broadly and finely regulate thyroid cell differentiation, growth and secretory functions (Cao et al., 2022). In a clinical study, 1800 patients with thyroid disease were divided into a thyroid nodule group and a non-nodule group, and serum levels of inflammatory factors such as IL-6 and TNF-α were measured, and regression analysis showed a positive correlation between thyroid nodules and IL-6 and TNF-α (Sun et al., 2016). The results of this study showed that the mRNA and protein levels of inflammatory factors (IL-6, TNF-α, IL-1β) were significantly elevated in the thyroid tissues of the thyroid nodules model rats, which was consistent with the clinical results, while JJJG was able to reduce the expression of IL-6, IL-1β, and TNF-α at both the gene and protein levels, which resulted in a good therapeutic effect.

Overexpression of inflammatory factors can exacerbate disease damage by inducing activation of the JAK2/STAT3 signaling pathway (Zhou et al., 2022; Jiang et al., 2023). Inflammatory factors bind directly to JAK2 and contribute to the phosphorylation of the JAK2 protein, which in turn activates the phosphorylation of the STAT3 protein and thus exert its specific biological function (Zhong et al., 2021). JJJG was able to reduce the expression of inflammatory factors as well as the mRNA expression of JAK2 and STAT3, and decrease the levels of p-JAK2/JAK2 and p-STAT3/STAT3 to inhibit the activation of the JAK2/STAT3 pathway, thus effectively reducing the damage of thyroid tissue. Studies have shown that STAT3 has a binding site in the promoter region of the VEGF gene, and that activated STAT3 upregulates VEGF expression and promotes angiogenesis (Fossey et al., 2011). The findings of the experiment also verified the conclusion that activation of STAT3 and high expression of VEGF were present in the thyroid tissues of rats in the model group, and JJJG was able to reverse this alteration, resulting in good therapeutic effects.

In the clinical study, serum VEGF levels were significantly elevated in patients with thyroid nodules and decreased after oral treatment with Bei Mu E Xiao Pills, suggesting that Bei Mu E Xiao Pills may downregulate VEGF levels in order to reduce the size of nodules (Liu et al., 2015). The clinical study result is consistent with the phenomena in our present study. VEGF levels in thyroid tissue of model group were significantly increased. 6 weeks’ continuous administration of JJJG markedly attenuated thyroid nodules through suppressing the mRNA and protein expression of VEGF. These results indicated that abnormal VEGF gene and protein expression was closely related to the pathogenesis of thyroid nodules and is also an important target for drug therapy. Besides, in order to investigate the involvement of angiogenesis in the pathological process of renal fibrosis, the distribution of VEGF expression in the kidney was examined by immunofluorescence, and the results showed that VEGF was mainly highly expressed in the renal tubular epithelial cells of the model rats (Yang et al., 2021). In concert with these findings, we similarly found that VEGF was highly expressed in follicular epithelial cells of rats in the thyroid nodules model.

Taken together, we demonstrated that JJJG has good therapeutic effect on thyroid nodules and is a promising external preparation for the treatment of thyroid nodules. Meanwhile, this study is the first to explore and suggest that the (IL-6, TNF-α, IL-1β)/JAK2/STAT3/VEGF signaling pathway may be involved in regulating the development of thyroid nodules and is one of the possible therapeutic targets for JJJG. However, further research is needed to validate the action mechanism of JJJG by adding inhibitors or agonists of the critical pathway in cell experiments *in vitro*. Furthermore, due to constraints imposed by the COVID-19 pandemic and the stepwise nature of our study design, mRNA sequencing was not performed on human samples. Future research will prioritize the collection of patient samples with confirmed thyroid nodules diagnoses. These samples will then be subjected to mRNA sequencing and subsequent experimental validation. This strategy will not only explore the therapeutic mechanism of JJJG from the perspective of authoritative clinical studies, but also validate the accuracy of animal experimental methodology, thus laying a solid scientific foundation for future research.

## Conclusion

**FIGURE 13:**
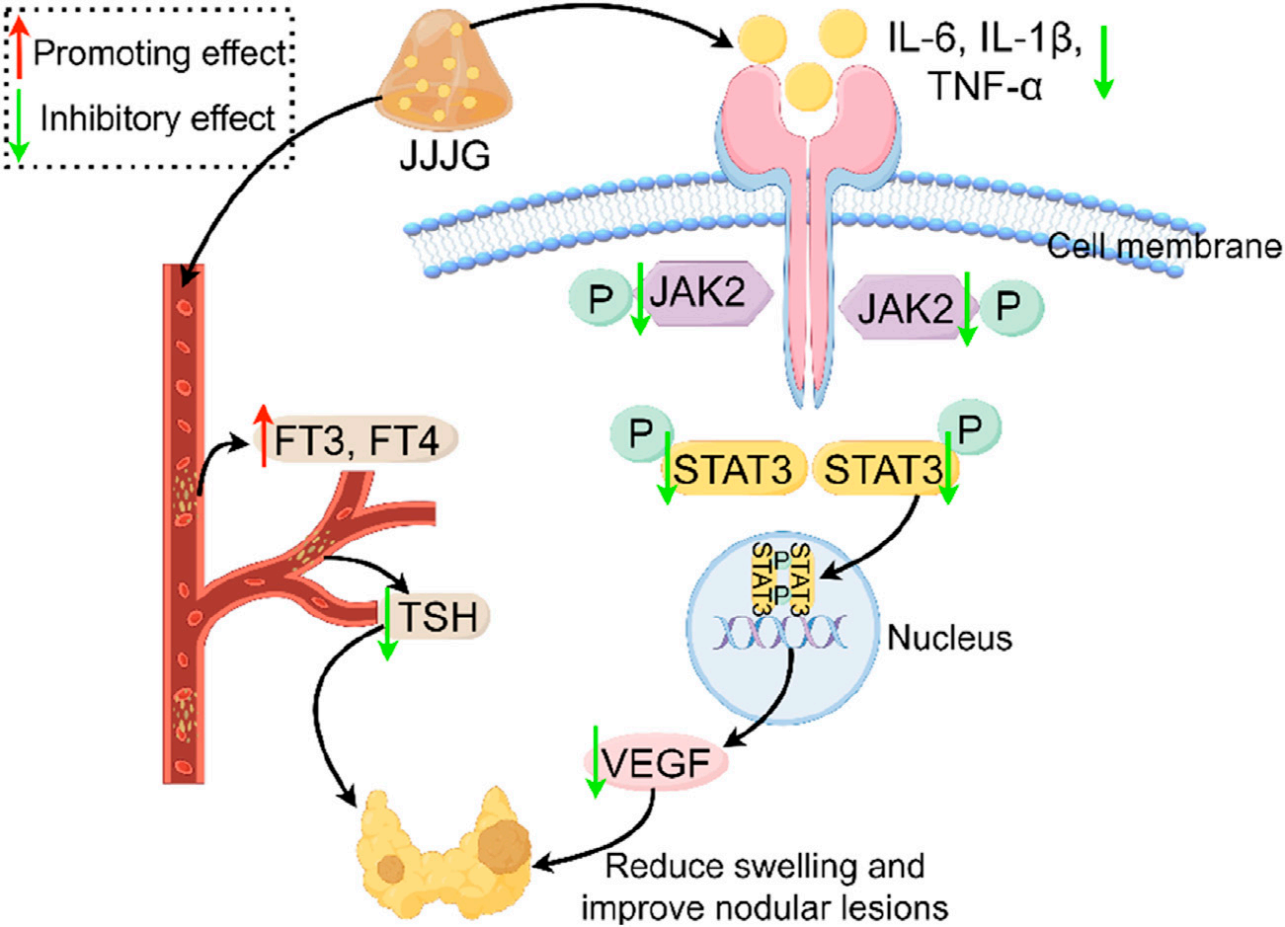
Mechanism of thyroid nodules treated with JJJG. JJJG modulated the levels of FT3, FT4 and TSH in serum and reduced inflammatory cytokines, inhibited the activation of JAK2/STAT3 pathway, and downregulated VEGF expression to ameliorate thyroid nodules.

The whole results confirmed that JJJG can reduce swelling and improve nodule lesions, thus effectively treating thyroid nodules by regulating FT3, FT4 and TSH levels in serum, simultaneously reducing inflammatory factors in thyroid tissue, inhibiting the activation of JAK2/STAT3 signaling pathway, and reducing VEGF expression (Figure 13).

## Data Availability

The raw data presented in the study are deposited in the FigShare repository, available at https://doi.org/10.6084/m9.figshare.27192723. Further inquiries can be directed to the corresponding authors.
